# Analyzing pediatric forearm X-rays for fracture analysis using machine learning

**DOI:** 10.1007/s11548-025-03485-z

**Published:** 2025-07-24

**Authors:** Van Lam, Abhijeet Parida, Sarah Dance, Sean Tabaie, Kevin Cleary, Syed Muhammad Anwar

**Affiliations:** 1 Sheikh Zayed Institute for Pediatric Surgical Innovation, Children’s National Hospital, Washington, DC USA; 2 School of Medicine and Health Sciences, George Washington University, Washington, DC USA; 3 Nationwide Children’s Hospital, Columbus, OH USA

**Keywords:** Machine learning, Self-supervised learning, Pediatric orthopedics, Forearm fractures

## Abstract

**Purpose:**

Forearm fractures constitute a significant proportion of emergency department presentations in pediatric population. The treatment goal is to restore length and alignment between the distal and proximal bone fragments. While immobilization through splinting or casting is enough for non-displaced and minimally displaced fractures. However, moderately or severely displaced fractures often require reduction for realignment. However, appropriate treatment in current practices has challenges due to the lack of resources required for specialized pediatric care leading to delayed and unnecessary transfers between medical centers, which potentially create treatment complications and burdens. The purpose of this study is to build a machine learning model for analyzing forearm fractures to assist clinical centers that lack surgical expertise in pediatric orthopedics.

**Methods:**

X-ray scans from 1250 children were curated, preprocessed, and manually annotated at our clinical center. Several machine learning models were fine-tuned using a pretraining strategy leveraging self-supervised learning model with vision transformer backbone. We further employed strategies to identify the most important region related to fractures within the forearm X-ray. The model performance was evaluated with and without region of interest (ROI) detection to find an optimal model for forearm fracture analyses.

**Results:**

Our proposed strategy leverages self-supervised pretraining (without labels) followed by supervised fine-tuning (with labels). The fine-tuned model using regions cropped with ROI identification resulted in the highest classification performance with a true-positive rate (TPR) of 0.79, true-negative rate (TNR) of 0.74, AUROC of 0.81, and AUPR of 0.86 when evaluated on the testing data.

**Conclusion:**

The results showed the feasibility of using machine learning models in predicting the appropriate treatment for forearm fractures in pediatric cases. With further improvement, the algorithm could potentially be used as a tool to assist non-specialized orthopedic providers in diagnosing and providing treatment.

## Introduction

Among pediatric cases, forearm fractures constitute a significant proportion of emergency department presentations in the USA, with an estimated 5.5 million visits attributed to fall-related upper extremity fractures in the pediatric population between 2001 and 2015 [1, 2]. The primary diagnostic approach involves physical examination and radiography. The treatment goal is to restore length and alignment between the distal and proximal bone fragments. While minimally displaced fractures may necessitate only immobilization for comfort through splinting or casting, moderately or severely displaced fractures often require reduction for realignment. Depending on the characteristics of fractures, the treatments are mainly divided into three types including 1) non-reduction types, 2) reduction types, and 3) surgery [3, 4]. The treatment decision must be judiciously made, considering the specific characteristics and location of the fracture, as well as patient demographics.

Parents often take their children with suspected fractures to adult-based or urgent care medical centers, which usually lack the resources required for specialized pediatric care. This leads to transfers to pediatric tertiary care centers for urgent consultations from pediatric orthopedic surgeons. Another investigation from Practice Management Committee of the Pediatric Orthopedic Society of North America and American Academy of Pediatrics underscored that more than 50% of referrals to pediatric orthopedic surgeons could be effectively managed by primary care providers [5, 6]. In another study, it was demonstrated that the cost of the emergency room visit with attempted reduction was 50% more than splinting with early referral (US dollars 536 versus US dollars 270) [7]. A study showed that more than a quarter (28.7%) of all transfers did not have any additional diagnostic work-up and/or therapeutic intervention in the PED and were discharged home in 12 h or less after transfer. The annual total charges calculated for these pediatric transfers in our study were US $489,143 [8]. The high incidence of frequent referrals and transfers from primary care, urgent care, and emergency department to specialized centers can cause unnecessary transfer, potential delay, and treatment complications and create time, labor, and financial burdens on the hospitals, patients, and their families.

Despite the existence of various artificial intelligence (AI)-assisted fracture recognition algorithms, current algorithms predominantly focus on detecting (classification or region of interest detection) fractures [9, 10]. There is a notable absence of models leveraging this information to predict the optimal treatment for identified fractures [9, 10]. A deep-learning system's proficiency in detecting fractures in adult musculoskeletal radiographs was evaluated, reporting an impressive sensitivity of 95.3% and specificity of 81.3% [11]. GRAZPEDWRI-DX dataset was specifically curated for automatic pediatric wrist fracture detection [12]. A recent study showed the feasibility of the machine learning model in assisting pediatric orthopedics with quantifying migration percentage (MP) value from hip X-ray scans [13]. However, automated analysis of pediatric forearm fractures using radiology imaging remains unexplored largely due to non-availability of pediatric specific datasets.

Recently self-supervised methods using transformers have seen widespread success in computer vision applications [14]. Self-supervised learning (SSL) is an alternative to supervised learning when it is hard to obtain labels (ground truth) for large-scale dataset. The fundamental idea is to generate supervisory signals from the unstructured data by defining a pretext task, where a machine learning framework learns the underlying structure of the data to solve the task. To that end, SSL methods can be trained using much bigger data that is less affected by human labeling bias and noise within the data [15]. A group masked model learning (GMML) paradigm was developed for knee abnormality classification using MRI data. The method, based on transformer, performed better than state of the art when trained on small data (~ 1000 subjects) [16]. Group masked model learning is used as a pretext task for pretraining the transformer weights. The images (forearm X-rays in our case) are modified by introducing random noise and replacing patches with zeros generating the corrupted image. The task for the model is to reconstruct the corrupted image. Hence using GMML, the model is trained to learn the underlying concepts in the image. Once GMML training concludes, the model weights are used to perform downstream tasks using fully supervised training with actual labels (reduction, no-reduction in our case). In a chest X-ray study, the masked image modeling paradigm was extended by adding contrastive loss to learn meaningful representation in the self-supervised pretraining stage. The trained model was able to perform multiple tasks, including classification (COVID/pneumonia/healthy) and lung segmentation for both pediatric and adult subjects [17]. GMML is built using transformers, which are neural networks with built in attention mechanism and have recently been used in state-of-the-art methods for image analysis and natural language processing [18–20]. Vision transformers process a sequence of patches representing tokens of the input forearm x-ray image x ∈ R H × W. These are flattened to 2D patches (p × p pixels), where H and W are the height and width of the input image. Building on the success of these studies, in this work, we developed an automated framework that gives treatment recommendations for pediatric fractures using X-ray images of wrist or forearm. Our contributions include:

1) We developed a pretraining model using self-supervised learning with vision transformer with public dataset containing X-rays.

2) We perform fine-tuning of the pretrained model with our in-house dataset (labeled) to classify the treatment recommendation, which are “no-reduction needed” (class 0) and “reduction needed” (class 1).

## Methods

### Data curation

We use three different public datasets including MURA [21], FracAtlas [22] and UNIFESP [23]. These datasets contain normal and abnormal X-ray scans of different parts of the body such as shoulder, elbow, humerus, forearm, hand, and wrist. There are 40,561, 4,083, and 2472 scans from MURA, FracAtlas, and UNIFESP, respectively, bringing the total number of images to 47,116. Our in-house dataset from the Children’s National Medical center includes 1250 children who were presented to the Hospital or were transferred from outside institutes for forearm fracture treatment. Patients from 0 to 18 years old, all genders, and ethnicities who were identified with having forearm fractures are included in the study. Patients with elbow fractures or dislocations are excluded from the study, since such cases will be directly referred to surgery. Data were curated with the help of an expert orthopedic surgeon and clinical associates. The data were collected for patients who were seen at our clinical center between 2015 and 2020. Mean age was 7.98 with population standard deviation of 3.87. In 1250 children, 789 fractures on ulna bone and 1192 fractures on radius bone were identified. Anterior posterior (AP) and lateral (LAT) views of each patient’s X-ray scans were downloaded from our hospital’s picture archiving and communication system (PACS) in DICOM format, anonymized, resized to 512 by 512 pixels, and stored as JPEG images. Overall, the curated dataset includes 2404 images, with 1202 scans for AP view and 1202 scans for LAT view. Training, validation, and testing were divided into 60%, 20%, and 20%, respectively, in both pretraining and fine-tuning models.

### Manual and automated annotations

**Fig. 1:**
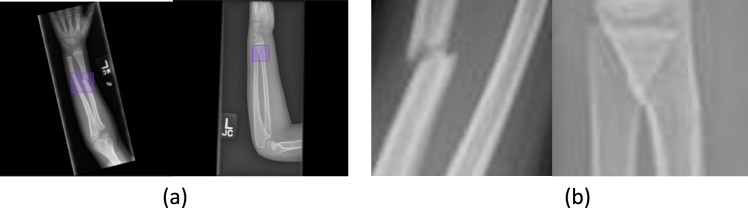
Forearm fractures annotation. a) Manual bounding box annotations and (b) crops from YOLO identification

A pediatric surgeon manually annotated the fracture area of 300 images of CNH dataset (150 images for AP view and 150 images LAT view) by drawing a box using a web-based tool called “makesense.ai”[24]. The coordinates of the box of each image were exported and stored in a text file. Figure 1a (left) shows a displaced fracture on radius bone, and Fig. 1a (right) shows a buckle fracture on radius bone. Recently, you only look once (YOLO) is popular in the field of computer vision for effective object and region of interest (ROI) bounding box detection [25]. The total of 300 images (150 AP and 150 LAT images) were manually annotated to identify the ROI by our team and were used to fine-tune the YOLOv11 for fracture ROI detection. We then used the fine-tuned YOLOv11 to make predictions on the remaining 2104 images. Figure 1b illustrates the fracture areas detected by YOLO identification on the same cases as shown in Fig. 1a.

### Self-supervised Model Training

**Fig. 2:**
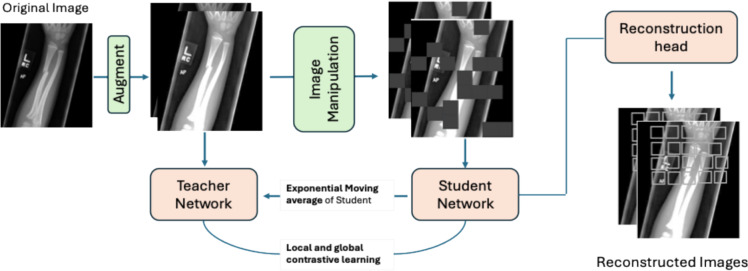
Our proposed self-supervised learning strategy for forearm X-rays

We leverage a pretraining strategy using self-supervised learning model (Fig. 2) that uses vision transformers to learn the normality and abnormalities in the scans without any labels and to identify the features of fractures. This model design incorporates a Vision Transformer (ViT) architecture in a student–teacher framework to achieve superior feature representation learning by leveraging local and global contrastive loss as well as a reconstruction head for input image reconstruction. We used rotation, cropping, and horizontal flipping for image augmentation. Image manipulation was performed using GMML [16]. The model input during the pretraining stage was X-rays from the public dataset, whereas, for fine-tuning, the input to the model was a three-channel image where the first channel is AP view, second channel is LAT view, and third channel is a zero channel.

## Experiments and results

### Pretraining model using self-supervised learning with vision transformer

The pretraining stage leverages the public datasets with the task to reconstruct masked input images. The model was trained for 2000 epochs with a batch size of 32 on a computer with one Nvidia RTX A5000 with VRAM 24 GB. The lowest training loss was 0.05, which occurred at epoch 1880, and the model weights from this stage were used for subsequent experiments.

### Fine-tuning the pretrained model

The pretrained model was then fine-tuned to classify the treatment recommendations, including *“no-reduction needed”* (class 0) which represents patients only needing cast or splint for immobilization or *“reduction needed”* (class 1) which represents cases where reduction is required before performing any types of immobilizations. We implemented two fine-tuning model training strategies: 1) fine-tuning with original images from CNH dataset (total 1017 images, 344 images for “no reduction needed”—class 0 and 673 images for “reduction needed”—class 1). 2) Fine-tuning with ROI crops from CNH dataset (total 1017 images, 344 images for “no reduction needed”—class 0 and 673 images for “reduction needed”—class 1).

Cross-entropy was used as a loss function for model training and validation. Our performance metrics included sensitivity (TPR), specificity (TNR), area under the receiver operating curve (AUROC), and area under the precision–recall curve (AUPR). TPR measures the proportion of actual “reduction needed” cases and TNR measures the proportion of actual “no-reduction needed” cases that were correctly identified by the models. AUROC plots the true-positive rate (sensitivity) against the false-positive rate (1-specificity) at different classification thresholds, and a value closer to 1 demonstrates higher performance. AUPR represents the model performance in terms of precision and recall and is a widely used metric for imbalanced datasets. A higher value of AUPR indicates the model has good precision and recall, which is particularly important for imbalanced datasets [26]. Table 1 shows the TPR, TNR, AUROC, and AUPR values of the five fine-tuned models at 200, 400, and 800 epochs. The fine-tuned model used regions cropped with ROI identification output in the highest TPR and TNR of 0.83 and 0.74, respectively. The value of AUROC of 0.81 shows the model’s ability model in distinguishing “no-reduction need” and “reduction needed” cases. The AUROC *p*-values of 0.0047 and 0.00044 when incorporating ROI for 400 and 800 epochs compared with training “without ROI at 200 epochs” show that using ROI significantly improves the performance (*p* < 0.05). AUPR scores at 0.87 emphasize the strong performance of the model despite the imbalance between the two classes. The p-values were calculated using DeLong’s test.

**Table 1:** Results for various performance metrics used for model evaluation

ROI (using YOLO)	Number of epochs trained	Training time	True positive rate (TPR)	True Negative Rate (TNR)	AUROC	AUPR	*p*-values using DeLong’s method
No	200	16 min	0.88	0.27	0.66	0.78	–
No	400	30 min	0.87	0.34	0.64	0.77	0.51
No	800	60 min	0.82	0.33	0.67	0.80	0.69
Yes	400	30 min	0.84	0.57	0.79	0.86	0.0047
Yes	800	60 min	0.83	0.74	0.81	0.87	0.00044

## Discussion

There are several studies that have focused on automated fracture detection on different body parts using convolutional neural network. For instance, convolutional neural networks-based models were widely used to automate the detection of supracondylar fracture [27], proximal humerus fracture [28], or intertrochanteric hip fracture [29]. A meta-analysis suggests that AI can be used to enhance diagnostic performance in fracture detection, showing promise as a useful diagnostic tool [30]. However, there is a gap in terms of having reliable clinical tools for fracture management. Hence, to overcome challenges in providing timely, accurate treatment for patients, avoiding delays and unwanted transfers, a treatment recommendation tool is essential. As our goal is to assist non-orthopedic healthcare providers in determining optimal treatment, TPR and TNR are two key factors in determining the accuracy of the algorithm. The decision of “reduction needed” or “no-reduction needed” for treating fracture is based on several factors such location of the fractures, fracture types (buckle, transverse, and oblique), type of displacement (non or minimally angulated, shortened, translated, or rotated). The consequences of missing a fracture that needs reduction creates persistent pain, stiffness, and discomfort for patients. In the worst scenario, it can lead to lasting complications that might require surgery later to re-align the bone fragments. On the other hand, when a fracture does not require reduction but is diagnosed as a “reduction needed” case, the patient must be transferred to orthopedics. This process can cost time, labor and financial burdens that create heightened stress and fatigue for patients, their families as well as orthopedics. For “reduction needed” cases, fractures are moderately or severely angulated, shortened, translated, or rotated, which are easier to identify. For “no reduction needed” cases, the fractures are usually non-displaced or minimally displaced, which are harder to identify and sometimes can be treated differently from clinicians to clinicians. Therefore, the chance of missing a fracture that needs reduction is lower than diagnosing “no-reduction needed” case to be “reduction needed” and transfer to orthopedics.

It is illustrated that without YOLO incorporated, TPR value at 800 epochs is slightly different (0.82 versus 0.83) but TNR is significantly lower (0.33 versus 0.74) (Table 1). With AUROC p-values of 0.0047 and 0.00044, it is emphasized that ROI identification plays a major role in distinguishing the two classes “no-reduction needed” and “reduction needed.” Our pretraining model, using self-supervised training paradigm, utilized fracture data from different public dataset and hence will ensure that the trained models are robust and generalized to a wide range of conditions and patient populations. The datasets represent a broad spectrum of cases covering most of the body parts. MURA has binary labels representing normal and musculoskeletal abnormality. FracAtlas represents X-rays with fractures/no-fractures in different body parts and UNIFESP represents X-rays with labels for different body parts. Hence, our training data are diverse in terms of body parts as well as clinical conditions. This would ensure the training generalizes to various conditions.

Although the proposed methodology achieves promising results, we acknowledge that there are still limitations. Our dataset size is relatively small and imbalanced as the number of images for “class 1” is approximately double when compared to “class 0.” Hence, in future extension of this study we will incorporate data from multiple clinical centers and improve the ROI identification method. This will also help with optimized and faster data labeling.

## Conclusion

We presented a method to assist non-orthopedic healthcare providers in treating pediatric fractures without necessitating consultation with a pediatric orthopedic surgeon. Overall, the model performed better when fine-tuned with regions cropped from the forearm X-ray using ROI identification. When training for 800 epochs, the model demonstrated an AUROC and AUPR of 0.81 and 0.87, respectively. We can clearly see that the model did not perform well when predicting “no-reduction needed” cases when ROI was not incorporated, as evident by the TNR value (0.33 compared to 0.74 with ROIs). Overall, the method shows promising performance in identifying the correct treatment by analyzing the forearm X-rays. These results could help in building a clinical recommendation system for triaging and identifying cases that need immediate attention, extending this capability to clinics lacking surgical expertise in pediatric orthopedics.
